# The Role of Antiepileptic Treatment in the Recurrence Rate of Seizures After First Attack: A Randomized Clinical Trial

**Published:** 2015

**Authors:** Farhad ASSARZADEGAN, Hanif TABESH, Omid HESAMI, Hojjat DERAKHSHANFAR, Nahid BELADI MOGHADAM, Arya SHOGHLI, Andrew David BEALE, Seyed-Mostafa HOSSEINI-ZIJOUD

**Affiliations:** 1 Neurology Department, Emam Hossein Medical and educational Center, Shahid Beheshti University of Medical Sciences(SBMU), Tehran, Iran; 2 School of Medicine, Shahid Beheshti University of Medical Sciences (SBMU), Tehran, Iran.; 3 Emergency Department, Shahid Beheshti University of Medical Sciences (SBMU), Tehran, Iran; 4 Circadian Biologist, London, United Kingdom; 5 Social Development and Health Promotion Research Center, Kermanshah University of Medical Sciences, Kermanshah, Iran

**Keywords:** Epilepsy, Recurrence rate, Seizure, Sodium valproate

## Abstract

**Objective:**

Epilepsy is a serious, potentially life-shortening brain disorder that occurs in patients of all ages and races. A total of 2–4% of people have experienced seizures at least once in their lifetime. Although treatment usually begins after a seizure, it is an important question whether the first cases of seizure do need to be treated by antiepileptic drugs. In this manner, we compare the recurrence rates of epilepsy in first seizure patients treated with sodium valproic acid as an antiepileptic drug versus a placebo.

**Material & Methods:**

In a randomized clinical trial study, 101 first seizure patients were randomly divided into two groups: one group was treated with antiepileptic drugs (sodium valproate 200mg, three times a day) and the other group was given a placebo. The recurrence rate of seizures was evaluated and compared between the groups after 6 months of follow up.

**Results:**

Eight recurrence cases were detected. All recurrence cases came from the placebo group, with four patients suffering an additional seizure after four months and between 4-6 month follow up. A comparison of recurrence rate detected a statistically significant difference between the drug group and placebo group.

**Conclusion:**

Our data shows that the recurrences occurred only in the placebo group with the difference between the recurrence rates in the placebo versus drug-treated was significant. Our results suggest that drug therapy for people after their first seizure attack might reduce the probability of seizure recurrence.

## Introduction

Epilepsy is one of the most common neurological disorders in the world with more than 50 million affected. The incidence rate of epilepsy in the general population is 0.2–0.4%. Researchers have shown that in developing countries, 2–4% have recurrent epileptic attacks during their lifetime (1). However, because of the side effects and cultural issues due to antiepileptic drugs, some patients refuse to use antiepileptic drugs. Considering the high incidence of convulsions and the complications of uncontrolled seizures, it is a priority to control this disorder. The mortality and morbidity rates increase in recurrent cases (1). There is controversy regarding treatment of first time seizure patients since they are not defined as having epilepsy after just one seizure. In order to make a decision in these cases, recurrence rates and the effectiveness of antiepileptic drugs need to be evaluated. First seizures are a terrifying experience. It has been shown that any person has a 8–10% chance of experiencing a seizure event and 3% chance of becoming epileptic (2). Most of the time, the cause of first seizure is a pathology in the central nervous system (CNS) after which the recurrence rate is 30–50%. This recurrence rate rises to 70–80% after the second unprovoked seizure making the diagnosis of epilepsy more probable (3-7). First seizures have important differential diagnoses including syncope (breathing cessation and loss of consciousness), transient ischemic attacks (TIA), metabolic encephalopathy (hypoglycemia, electrolyte imbalances), nightmares, complex migraine, cardiac arrhythmia, and pseudoseizures. Convulsive syncope is a new topic and is defined when syncope induces seizures or death. Precise history taking is crucial for diagnosis but there is often no single diagnostic finding in patient histories. Investigations demonstrate that most patients with tonic-clonic seizures have had a previous simple or complex partial seizure (2, 8, 9). About 25–30% of first seizures are symptomatic and provoked by a CNS pathology or electrolyte imbalances or metabolic disorders (10-13). Convulsion inducing factors are fever, head trauma, excessive alcohol consumption, withdrawal syndromes of alcohol or illicit drugs, cerebral infections, ischemic attacks, intracranial hemorrhage, and drugs, such as clozapine, maprotiline, tramadol, theophylline, and baclofen, after baclofen. Seizures induced by metabolic disorders or toxicities have a lower chance of becoming epilepsy (lower than 3%) but organic disorders like cerebral abscesses increase the chance to greater than 10%. Seizures occurring after physiological stresses are not categorized as “acute symptomatic” and are instead called “triggered seizures”. Laboratory data of complete blood test and urine and cerebrospinal fluid (CSF) analysis are needed to diagnose these patients (14). To avoid recurrent attacks, patients should be aware of inducing factors as the recurrence rate of a first seizure after two years is approximately 42% (15). The significance of two unprovoked convulsions in one day is still unclear and considering these convulsions as a single simple seizure is controversial (16). In a quantitative review, the risk of seizure recurrence near two years following first attack was 36% and 47%, in prospective and retrospective studies, respectively (15). To avoid recurrence, the recommendation is to avoid provoking activities after first seizure for three months (17). Driving restrictions after the first seizure are controversial and most neurologists believe that patients should avoid driving for 3 months. Antiepileptics are a class of drugs that suppress attacks of epilepsy or reduce their frequency and severity. Valproic acid, often called valproate, is one such antiepileptic with a double mechanism of action: it decreases the influx of sodium and increases that of chloride by indirect Gamma-Aminobutyric Acid (GABA) mimetic effect. By these two complementary mechanisms, valproic acid increases the polarization of the cell and decreases its excitability. Its GABAmimetic effect prevails on its effect on the voltagedependent sodium channels. It is effective in the majority of epilepsies and is widely used (5, 10, 12). The purpose of the current randomized clinical trial is to compare the recurrence rate of epilepsy in the first seizure patients treated with sodium valproate acid as an antiepileptic drug or placebo.

## Material & Methods

Patients referred to the Neurology Clinic of Emam Hossein Medical and Educational Center (from 2009– 2011) with a first seizure were evaluated with inclusion and exclusion criteria. In all patients, the first seizure was generalized tonic-clonic form (GTC). If the patient had a family history of seizures, history of head trauma, tumors, meningitis, and neurological disorders, they were excluded. A thorough neurologic examination, electroencephalography (EEG), and a magnetic resonance imaging (MRI) were then performed. If all of the examinations, the EEG, and the MRI were normal and the patients had not taken any specific drugs, they were then included in the project. Patients were randomly categorized into two groups. One group (n = 50) received sodium valproate 200 mg three times a day as an antiepileptic drug and the other group (n = 51) was given multivitamins as a placebo. Patients were followed for 6 months and evaluated for recurrence rate and drug side effects. Data were collected by filling in questionnaires and interviews. Regarding ethics, the patients in the placebo group were excluded if they experienced seizures during the follow up period and these patients received appropriate medicine. The data were analyzed using SPSS (v.19). Frequency tables and scattering parameters obtained using descriptive statistics and reported as mean ± SD. Qualitative variables were compared between the two groups using Chi-square test and quantitative variables analyzed by t-test. In this study, a significant level of p = 0.05 was considered. In accordance with the Helsinki Declaration and the Medical Ethics Committee of Shahid Beheshti University of Medical Sciences (SBMU), all patients signed a written informed consent form for this project.

## Results

A total of 101 patients were included in this research with 50 patients receiving antiepileptic drugs (sodium valproate group) and 51 receiving a placebo (placebo group). Fifty-seven patients were male (56.9%) and 44 were female (43.1%). The mean age of patients was 34.7 ± 16.3. Patient educational status was divided into 4 levels, 10 cases (9.9%) were illiterate, 22 (21.8%) with elementary education, 45 (44.6%) with diploma, and 24 (23.7%) with masters or above. The mean duration of first seizure was 4.9 ± 4.2 minutes. There was not a significant difference in demographic characters between the two groups (p > 0.05) (Table 1).

**Table 1:** Characteristics of 101 Patients in the Sodium Valproate & the Placebo Groups

**Data**		**Group **		**P value**
		**Sodium Valproate** **(n = 50)**	**Placebo** **(n = 51)**	
**Age **	Mean ± SD	33.6 ± 9.4	36.9 ± 7.7	0.19
**Gender**	Male (n)	27	30	0.4
	Female (n)	23	21	
**Educational status **	Illiterate (n)	5	5	0.28
	Elementary (n)	10	12	
	Diploma (n)	24	21	
	Masters or above (n)	11	13	
**Duration of first seizure**	Mean ± SD	5.2 ± 4.4	4.7 ± 3.9	0.61

**Fig 1:**
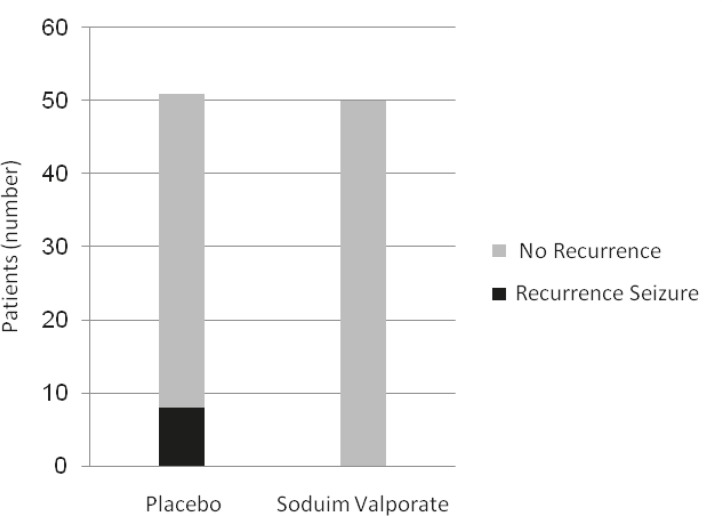
Seizure recurrence rates in both groups (sodium valproate 200 mg three times a

 Ninety-three patients (92.2%) did not have another seizure six months after the first seizure. Eight patients (7.8%) had recurrent seizures, of which four patients (3.9%) had seizures in the first four months. The other patients had their second seizure between 4–6 months of follow up. All of these 8 patients were in the placebo group (Figure 1). Analytical analysis shows a statistical significant difference in the recurrence rate between two groups (p = 0.04).

day and placebo group)

## Discussion

Giving anti-epileptic drugs after the first seizure is controversial (18-23). In previous pediatric research, it was concluded that antiepileptic drugs diminish the chance of epilepsy recurrence; however, they have no role in the course of main management. Others endorse considering the likelihood of future attacks and the side effects of the drug before recommending them for first seizures (19, 20). Drugs with minimal side effects, good patient compliance, high effectiveness, and few contraindications are good choices for treatment. Phenytoin and barbiturates are unsuitable as they may cause some neurologic or psychological disorders. For focal seizures carbamazepine, clobazam (especially in pediatrics), gabapentin, carbamazepine, lamotrigine, topiramate, and valproate are recommended. The last three drugs are also used in the management of general seizures. First seizures may lead to future complications depending on the risk factors. The chief complaint of 0.2–0.3% of emergency room (ER) patients is first seizures (24, 25). About 5% of the general population has at least experienced nonfebrile seizures in their lifetime (7, 26). Therefore, it is necessary to have a protocol for the treatment of this condition. The administration of the antiepileptic drugs after first attack is still controversial. Those supporting the use of antiepileptic drugs emphasize the importance of recurrence and prognosis of the patients. Supporting this, are researches that demonstrate that there is a 60–70% chance of recurrence after the first seizure in two years (15, 27-30). This rate is related to the primary etiology; if the cause is cerebral injury, head trauma, or other brain pathologies like tumors, then the risk of recurrence increases when compared to idiopathic seizures. Those against the use of the drugs are others who are concerned about the side effects of the drugs, especially on children. Musicco et al. prescribed carbamazepine, phenytoin, phenobarbital, or sodium valproate to treat the case group with first seizure and delayed treatment for controls. They showed that 24% of 215 patients in the case group and 42% of 204 patients in the control group experienced a second seizure. The most important predicting risk factors were age, benzodiazepine consumption, and an abnormal EEG. A total of 68% of patients treated with antiepileptic drugs immediately after first attack were symptom free in two years. Delayed treatment resulted in a higher recurrence rate (18). Marson et al. stated (19) that these statistically significant (p<0.05) lower recurrence rate after first seizure when given antiepileptic drugs compared to a placebo group these resukts are similar to our conclusions (Figure 1). However, some recent articles do not support our results. Lean et al. followed patients with first seizure. They treated the case group with antiepileptic medications and non-medical treatments for others. Their results indicated that early treatment of first seizure had no effect on prognosis and did not lead to a complete cure (26). Furthermore, Temkin et al. demonstrated that there is no effective drug to use after the first unprovoked seizures (31). Our study limitations were the number of cases, distinguishing between true seizures and seizure-like symptoms, and examining genetic disorders as an etiology of seizures. We recommend further multicentral studies using larger sample sizes, considering the exact etiology of first seizure, and a prolongation of follow up period to determine the drug of choice for treatment.

**In conclusion**, we conclude that there is a statistically significant reduction in the recurrence rate if the first time seizure patients are treated with antiepileptic medications without concern for the side effects of these drugs. Further investigations are needed on the choice of drug and the period of the treatment.
